# Elucidating the molecular pharmacology of trace amine‐associated receptor 1 to advance antipsychotic drug discovery

**DOI:** 10.1002/ctm2.1576

**Published:** 2024-02-05

**Authors:** Jingjing Yu, Zheng Xu, Wei Yan, Zhenhua Shao

**Affiliations:** ^1^ Division of Nephrology and Kidney Research Institute State Key Laboratory of Biotherapy, West China Hospital, Sichuan University Chengdu China; ^2^ Frontiers Medical Center, Tianfu Jincheng Laboratory Chengdu China

## 1 COMMENTARY

**FIGURE 1:**
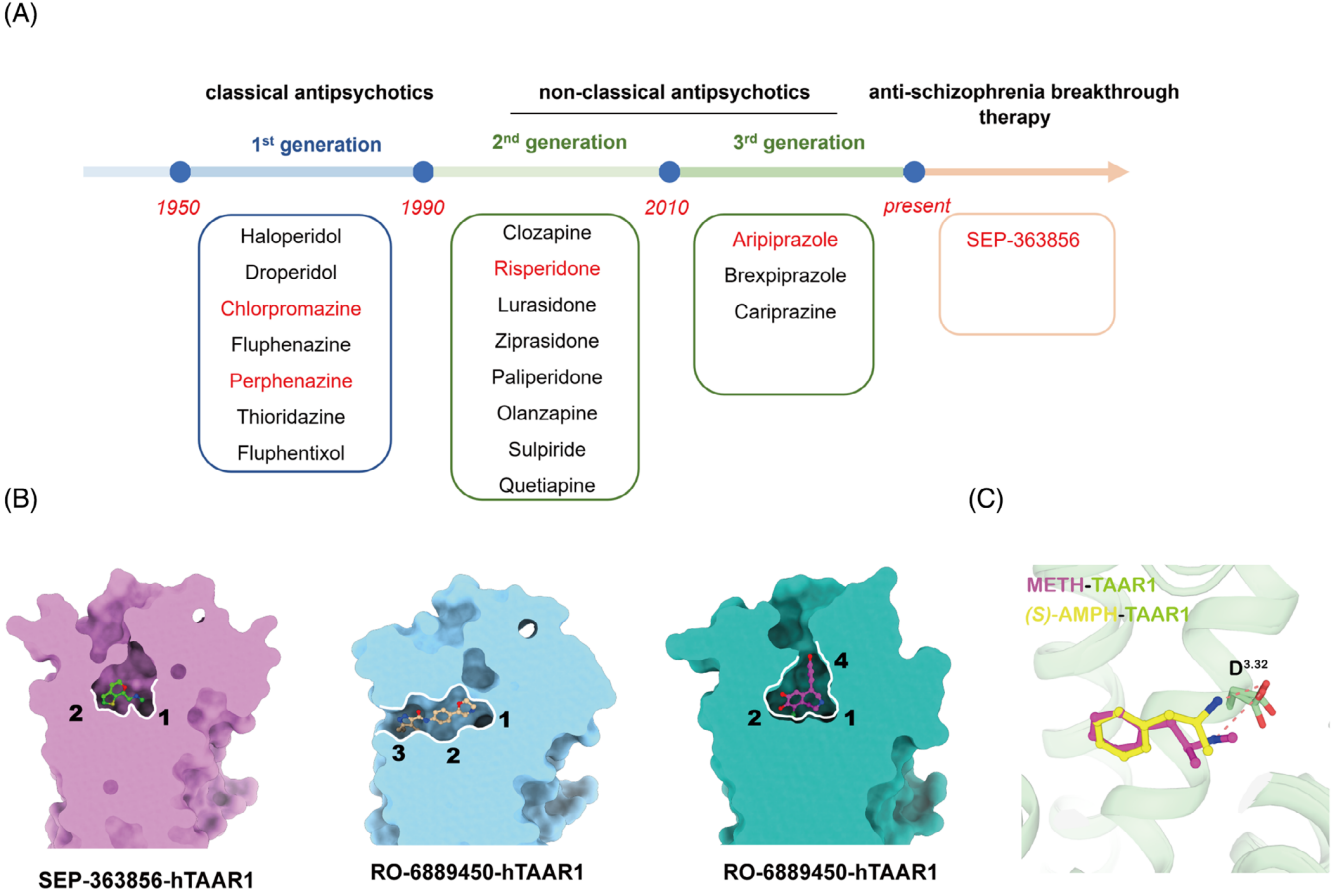
Mechanism of antipsychotic drugs targeting trace amine‐associated receptor 1 (TAAR1): (A) Representative of the development of antipsychotic drugs. (B) The plasticity of ligand recognition pockets of TAAR1 against different ligands such as SEP‐363856, RO‐6889450 and fenoldopam. (C) Methamphetamine (METH) and *(S)*‐AMPH show a similar conformation in binding to TAAR1.

Schizophrenia is a chronic mental disorder that influences the perceptions, emotions and behaviors of individuals, which can be categorized into three types: negative symptoms, positive symptoms and cognitive impairment.^1^, ^2^ Antipsychotics currently available on the market can be divided into two types: classical antipsychotics and non‐classical antipsychotics (Figure 1A).^1^, ^3^ Classical antipsychotics, such as chlorpromazine and perphenazine (Figure 1A), are potent dopamine D2 receptor (DRD2) antagonists that are effective in treating the positive symptoms of psychosis. However, they have limited efficacy against negative symptoms and are associated with a range of side effects.^1^ To enhance therapeutic outcomes, non‐classical antipsychotics such as risperidone have been developed that combine DRD2 antagonism with serotonin receptor activation (Figure 1A).^4^ This dual‐action profile not only contributes to the amelioration of negative symptoms but also carries a risk of metabolic side effects.^1^ However, current drugs are insensible to some positive patients as well as having limited or even ineffective efficacy on negative symptoms and cognitive functions.^1^, ^5^ Identifying novel therapeutic targets and developing new drugs are top priorities in the field of psychiatric research.

In the late 20th century, a class of amines was identified and named trace amines (TAs) due to their low concentrations (< 100 ng/g) at the tissue level.^5^ In 2001,^6^ TA‐associated receptors (TAARs), a G‐protein‐coupled receptor expressed in both the limbic and monoaminergic nervous systems of the brain, were found to be activated by these TAs, as well as by a range of psychoactive substances, such as amphetamines (AMPHs),^7^ modulating the central dopamine nervous system and serotonin neural circuits.^5^, ^8^, ^9^ Activation of TAAR1 inhibits midbrain dopaminergic and serotoninergic activity and enhances prefrontal glutamatergic neuron function, which has been associated with a variety of psychiatric disorders, including drug addiction, schizophrenia, and attention‐deficit hyperactivity disorder.^10^, ^11^ These pieces of evidence suggest that TAAR1 is a new high‐potential target receptor for the treatment of psychiatric disorders.

Recently, Roche developed a series of small molecules, including RO‐5256390, a TAAR1‐selective full agonist, and a partial agonist Ralmitaront (RO‐6889450), which exhibited antipsychotic, cognitive improvement and antidepressant effects in rodents (Figure 1A). Unfortunately, the monotherapy treatment of schizophrenia by ralmitaront did not show significant efficacy in phase II clinical trials (NCT04512066 and NCT03669640). Ulotaront (SEP‐363856),^12^ another small molecule antipsychotic drug targeting TAAR1 and 5‐HT_1A_R, developed by Sunovion Pharmaceuticals and PsychoGenics, was granted by the US Food and Drug Administration in 2019 as a non‐dopamine receptor‐targeted anti‐schizophrenia breakthrough therapy (Figure 1A).^13^ Although recently published clinical phase III results showed that it did not attain its therapeutic endpoints (NCT04072354), investigators believe that the results were influenced by COVID‐19 and additional phase III clinical trials are initiated and under recruitment state (NCT04825860 and NCT05359081). Despite TAAR1 being an attention‐grabbing receptor associated with psychosis recently, little has been reported on the molecular pharmacological properties of its ligands. Meanwhile, researchers reported that the pharmacological properties of drugs targeting human or rodent TAAR1 may be different.^14^ This ambiguous information severely hampers the translation of preclinical studies to clinical applications.

To address this issue, in late 2023, three articles were published in *Nature* and *Cell*, which systematically elucidated the molecular mechanisms and activation characteristics of TAAR1 in response to a set of ligands, including endogenous trace amines, and synthetic compounds, such as AMPH, methamphetamine (METH), ulotaront and ralmitaront.^15^, ^16^, ^17^

As the current TAAR1 clinical drug of interest, ulotaront could act on both TAAR1 and 5‐HT_1A_R but not dopamine receptors, yet, its mechanism of action (MOA) with TAAR1 and 5‐HT_1A_R is not clear. To this end, Xu et al.^17^ and Liu et al.^15^ independently resolved the cryo‐electron microscopy structures of ulotaront with TAAR1 and 5‐HT_1A_R, respectively. Liu et al. emphasized the MOA of ulotaront on the binding of two receptors, and additionally counted on the RO‐5256390‐bound TAAR1 structure to reveal the high‐selectivity mechanism of RO‐5256390 to TAAR1, which provides the basis for the design of novel drugs selectively targeting TAAR1.

While Xu et al. highlighted the similarities and differences in the molecular mechanisms of TAAR1 recognition by ulotaront and ralmitaront, proposed a distinctive binding mode for ralmitaront, and discovered a new ligand binding pocket of TAAR1 (Figure 1B). Xu et al. further screened and found that the catecholamines fenoldopam and A77636, which originally target the dopamine receptor,^18^ were also able to activate TAAR1. Based on the structural information of these two agents, another extended ligand‐binding pocket was identified. These results elucidate that TAAR1 exhibits a highly adaptable ligand recognition profile, composed of four principal binding sites, offering valuable insights for the design of drugs targeting TAAR1 (Figure 1B).^19^

Moreover, Shang et al.^16^ and Xu et al. found that many compounds, including ralmitaront, were able to activate the Gq and Gi signalling pathways via TAAR1. Shang et al. characterized the activation of various G protein signals downstream of TAAR1 by different endogenous and exogenous compounds in a systematic way, and verified that the Gs and Gq signalling is beneficial for the treatment of psychosis, while Gi signalling is the opposite. Based on that, Shang et al. went a step further by designing ZH8651, a small molecule agonist of TAAR1 with dual activation activity of Gs and Gq. This compound demonstrated a therapeutic effect on schizophrenia in a mouse model and surpassed ulotaront in a prepulse inhibition test experiment.

Notably, TAAR1 is also a target of AMPHs. Xu et al. and Liu et al. solved the structures of AMPH and METH with TAAR1 (Figure 1C), respectively, providing an important basis for the study of the molecular pharmacological mechanism of AMPHs. Additionally, the optical isomers of AMPH (*(S)‐*/*(R)*‐AMPH) exhibit species differences in targeting human TAAR1 (hTAAR1) and mice TAAR1 (mTAAR1) and vary in pharmacological activity toward the same drug, but the mechanism is unclear.^14^ Combined with structural analyses and signalling assays, Xu et al. elucidated the molecular mechanisms by which hTAAR1 and mTAAR1 recognize the two isoforms of AMPH, respectively, revealing the reason for the stronger therapeutic effects of *(S)*‐AMPH. Additionally, the study conducted a comparative analysis of the pharmacological responses of hTAAR1 and mTAAR1 to the same ligand, yielding significant insights that will inform the selection of suitable animal models for preclinical research.

Overall, these results, together with previous publications, collectively advance the understanding of the MOA of ligands targeting TAAR1, which could facilitate the development of TAAR1‐based antipsychotics, providing a solid and powerful boost to the development of a new generation of antipsychotics.

## AUTHOR CONTRIBUTIONS

Jingjing Yu, Zheng Xu, Wei Yan and Zhenhua Shao conceptualized and wrote the commentary.

## CONFLICT OF INTEREST STATEMENT

The authors declare no conflict of interest.

## ETHICS STATEMENT

Not Applicable.
